# Population Pharmacokinetics of Praziquantel in Pregnant and Lactating Filipino Women Infected with Schistosoma japonicum

**DOI:** 10.1128/AAC.00566-20

**Published:** 2020-08-20

**Authors:** Amaya L. Bustinduy, Ruwanthi Kolamunnage-Dona, Mark H. Mirochnick, Edmund V. Capparelli, Veronica Tallo, Luz P. Acosta, Remigio M. Olveda, Jennifer F. Friedman, William W. Hope

**Affiliations:** a Department of Clinical Research, London School of Hygiene & Tropical Medicine, London, United Kingdom; b Department of Biostatistics, University of Liverpool, Liverpool Health Partners, Liverpool, United Kingdom; c Department of Pediatrics, Boston University School of Medicine, Boston, Massachusetts, USA; d Department of Pediatrics, University of California, San Diego, La Jolla, California, USA; e Department of Epidemiology, Research Institute of Tropical Medicine, Manila, Philippines; f Department of Immunology, Research Institute of Tropical Medicine, Manila, Philippines; g Department of Pediatrics, Alpert Medical School of Brown University, Providence, Rhode Island, USA; h Center for International Health Research, Lifespan Hospital, Providence, Rhode Island, USA; i Antimicrobial Pharmacodynamics and Therapeutics, University of Liverpool, Liverpool Health Partners, Liverpool, United Kingdom; j Royal Liverpool, Broadgreen University Hospital Trust, Liverpool Health Partners, Liverpool, United Kingdom

**Keywords:** schistosomiasis, *Schistosoma japonicum*, pregnancy, lactation, breast milk, praziquantel, pharmacokinetics, PK

## Abstract

An estimated 40 million women of reproductive age are infected with one of three species of the waterborne parasite *Schistosoma* spp. Treatment with praziquantel (PZQ) via mass drug administration (MDA) campaigns is the mainstay of schistosomiasis control for populations living in areas of endemicity. The World Health Organization recommends that pregnant and lactating women be included in schistosomiasis MDA programs, and several recent studies have evaluated the safety and efficacy of PZQ use during pregnancy. To date, there are no data describing PZQ pharmacokinetics (PK) during pregnancy or among lactating postpartum women. As part of a randomized controlled trial investigating the safety and efficacy of PZQ during human pregnancy, we examined the PK of this therapeutic drug among three distinct cohorts of women infected with S. japonicum in Leyte, Philippines. Specifically, we studied the PK properties of PZQ among early- and late-gestation pregnant women (*n* = 15 each) and lactating postpartum women (*n* = 15) with schistosomiasis. We found that women in early pregnancy had increased apparent clearance and lower area-under-the-curve (AUC_0–24_) values that may be related to physiological changes in drug clearance and/or changes in oral bioavailability. There was no relationship between body weight and apparent clearance. The mean ± standard deviation partition ratio of plasma to breast milk was 0.36. ± 0.13. The estimated median infant PZQ daily dose would be 0.037 mg/kg of body weight ingested from breast milk, which is significantly lower than the dosage required for antischistosomal activity and not known to be harmful to the infant. Our PK data do not support the suggestion to delay breastfeeding 72 h after taking PZQ. Results can help inform future drug efficacy studies in pregnant and lactating women with schistosomiasis.

## Abstract (precis)

An estimated 40 million women of reproductive age are infected with one of three species of the waterborne parasite *Schistosoma* spp. Treatment with praziquantel (PZQ) via mass drug administration (MDA) campaigns is the mainstay of schistosomiasis control for populations living in areas of endemicity. The World Health Organization recommends that pregnant and lactating women be included in schistosomiasis MDA programs, and several recent studies have evaluated the safety and efficacy of PZQ use during pregnancy.

## TEXT

Over 240 million people are infected with one of three species of the waterborne parasite *Schistosoma* spp., including ∼40 million women of reproductive age. More than 700 million people are at risk of infection (1, 2). Schistosomiasis caused by the most common *Schistosoma* spp. (i.e., S. mansoni, S. japonicum, and S. haematobium) is responsible for 1.86 million disability adjusted life years (DALYS) (3). Schistosomiasis remains a significant cause of morbidity and mortality in countries of endemicity, despite the availability of praziquantel (PZQ), which is the only widely available antischistosomal drug (4). PZQ is a first-line agent for the control of schistosomiasis in populations living in areas of endemicity and is administered via mass drug administration (MDA) programs (4). Despite WHO endorsement of the inclusion of pregnant women in MDA programs, this is not necessarily practiced in many affected countries (5).

PZQ is orally bioavailable. Absorption is higher with carbohydrate- and fat-rich foods. PZQ undergoes significant first-pass metabolism and is predominantly cleared by oxidative mechanisms via cytochrome P3A4 (CYP3A4) and cytochrome P19A (CYP19A) (6). There is high interindividual pharmacokinetics (PK) variability, which is further exacerbated in individuals with liver disease (7). When PZQ was first licensed in 1979, it had not been formally studied in any pregnant or lactating women. PZQ is classified as a class B agent for use in pregnant women by the Food and Drug Administration (FDA). This classification is based on demonstrated safety in laboratory animal studies, but there is a lack of definitive data in humans. There is a paucity of information related to the PZQ PK in pregnant women (8) despite the high likelihood that the physiologic changes of pregnancy may affect PZQ absorption, distribution, and clearance (9). Physiological changes related to pregnancy may result in altered drug exposures, which may have an impact on the probability of therapeutic success (10).

In 2002 and 2006, the World Health Organization (WHO) recommended that all schistosomiasis-infected pregnant and breastfeeding women be treated with PZQ individually or during MDA programs (11). This was based on the expected accrued morbidity from schistosomiasis during cycles of pregnancy and lactation without treatment. These recommendations were made despite a lack of data describing PZQ pharmacokinetics in pregnancy and lactating women. Furthermore, there are no available data regarding the concentration of PZQ in human breast milk following maternal treatment to support the current recommendation to stop breastfeeding for 72 h after taking PZQ. Since that time, two randomized controlled trials (RCTs) (12, 13) support the safety of PZQ in pregnancy, and one (12) suggests a potential beneficial impact on the iron status of both the mother and infant (12). Although many countries have included pregnant and lactating women in MDA campaigns, many others are waiting for further data on the safety and PK of PZQ during pregnancy and lactation (5, 14).

As part of an RCT examining the safety and efficacy of PZQ during human pregnancy, we examined the PK of PZQ in pregnant and lactating women infected with S. japonicum living in the northeast of the province of Leyte, Philippines. The primary objective was to evaluate and compare the PK and safety of PZQ in early- and late-gestation pregnant women (*n* = 15 each) and in lactating postpartum women (*n* = 15) with schistosomiasis.

## RESULTS

### Study design and patient demographics.

**FIG 1:**
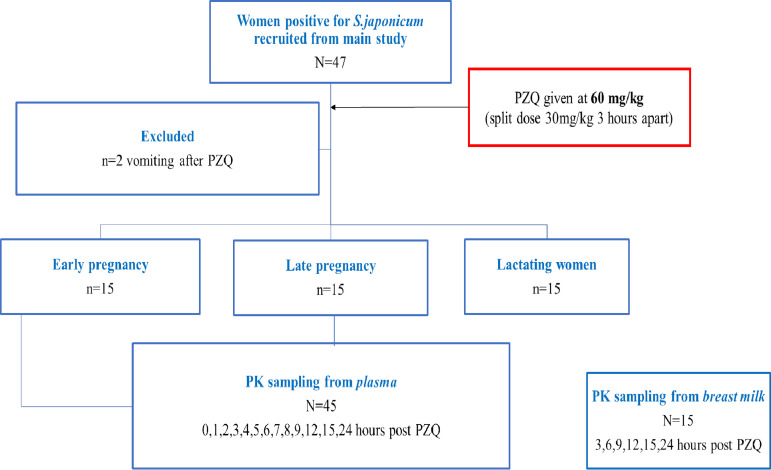
Study flow design.

The study design is shown in Fig. 1. A total of 47 women that were S. japonicum positive by parasitological examination were enrolled and received a PZQ split dose of 60 mg/kg of body weight, 3 h apart (i.e., two split dosages of 30 mg/kg) (12). Two patients in the early-pregnancy group vomited shortly after receiving PZQ and did not have PK sampling performed. This left a total of 45 patients who were divided evenly among the 3 groups: (ii) early pregnancy (i.e., 12 to 16 weeks gestation; *n* = 15); (ii) late pregnancy (i.e., 30 to 36 weeks gestation; *n* = 15); and (iii) lactating nonpregnant women (i.e., 5 to 7 months postpartum; *n* = 15). The weight and height for all women enrolled in the study are summarized in Table 1.

**TABLE 1:** Characteristics of the patients at enrollment

Characteristic	Data for study groups			
	Early pregnancy (*n* = 17)	Late pregnancy (*n* = 15)	Postpartum (*n* = 15)	All (*n* = 47)
Weight (kg) (mean [SD])	47.6 (8.12)	51.5 (7.08)	46.6 (7.40)	48.5 (7.69)
Height (cm) (mean [SD])	152.0 (5.45)	149.8 (5.63)	152.3 (4.56)	151.4 (5.24)
Age (yrs)				
Mean (SD)	23.8 (5.98)	26.5 (6.61)	26.5 (6.65)	25.5 (6.39)
Median	21.0	25.0	24.0	24.0
Minimum, maximum	18, 37	18, 37	20, 44	18, 44
Ethnicity (%)				
Non-Hispanic or non-Latino	17 (100.0)	15 (100.0)	15 (100.0)	47 (100.0)
Hispanic or Latino	0 (0)	0 (0)	0 (0)	0 (0)
Race (%)				
American Indian/Alaskan Native	0 (0)	0 (0)	0 (0)	0 (0)
Asian	17 (100.0)	15 (100.0)	15 (100.0)	47 (100.0)
Hawaiian/Pacific Islander	0 (0)	0 (0)	0 (0)	0 (0)
Black/African American	0 (0)	0 (0)	0 (0)	0 (0)
White	0 (0)	0 (0)	0 (0)	0 (0)
Multiracial	0 (0)	0 (0)	0 (0)	0 (0)
No. of prior live births (%)				
0	6 (35.3)	4 (26.7)	1 (6.7)	11 (23.4)
1–5	10 (58.8)	9 (60.0)	11 (73.3)	30 (63.8)
6–10	1 (5.9)	2 (13.3)	3 (20.0)	6 (12.8)
>10	0 (0.0)	0 (0.0)	0 (0.0)	0 (0.0)
Current smoking status (%)				
No	17 (100.0)	15 (100.0)	13 (86.7)	45 (95.7)
Yes	0 (0.0)	0 (0.0)	2 (13.3)	2 (4.3)
Current alcohol consumption (%)				
No	5 (29.4)	2 (13.3)	2 (13.3)	9 (19.1)
Yes	12 (70.6)	13 (86.7)	13 (86.7)	38 (80.9)
Intensity of S. japonicum infection (%)				
Low (<100 eggs per gram of stool)	16 (94)	15 (100)	15 (100)	46 (97.8)
Moderate (100–399 eggs per gram of stool)	1 (6)	0 (0)	0 (0)	1 (2.2)
Heavy (≥400 eggs per gram of stool)	0 (0)	0 (0)	0 (0)	0 (0)

### Population PK of PZQ in plasma.

**FIG 2:**
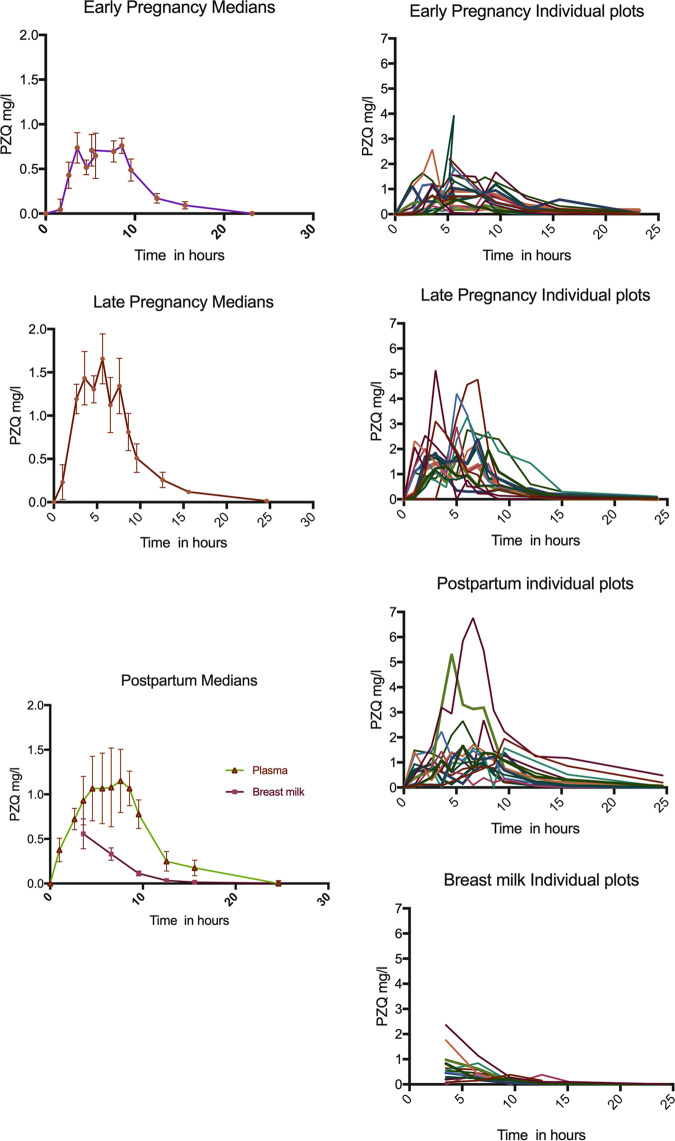
Median and individual PZQ concentration time profiles.

A population methodology was used to fit a structural PK model to the overserved plasma concentration-time data to enable robust estimates of interpatient variability. The median and individual PZQ concentration-time profiles for each study group are shown in Fig. 2. There was marked variability in the PZQ concentrations in both plasma and breast milk in all study groups. The fact that the dosing of PZQ was split, 3 h apart, resulted in more than one peak concentration for each patient.

**FIG 3:**
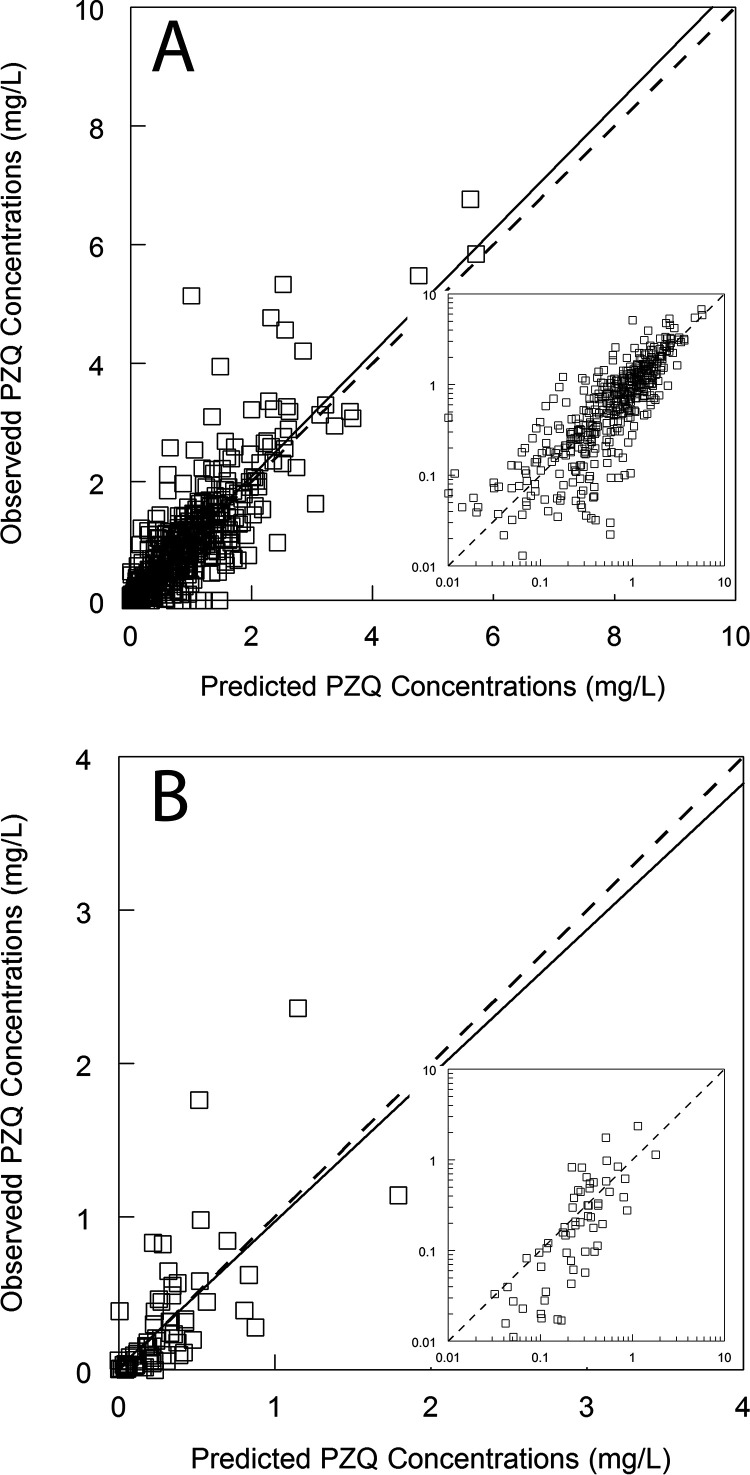
(A and B) The observed-predicted plots for the PZQ concentrations in plasma (A) and breast milk (B) after the Bayesian step. The median parameter values for each patient have been used. The observed-predicted data are plotted on a log-log plot for both outputs and are shown in the inserts. The regression line for plasma in panel A is given by observed = 0.016 + 1.04 · predicted; *r^2^* = 0.604. The regression line for breast milk in panel B is given by observed = 0.015 + 0.953 · predicted; *r^2^* = 0.468.

**FIG 4:**
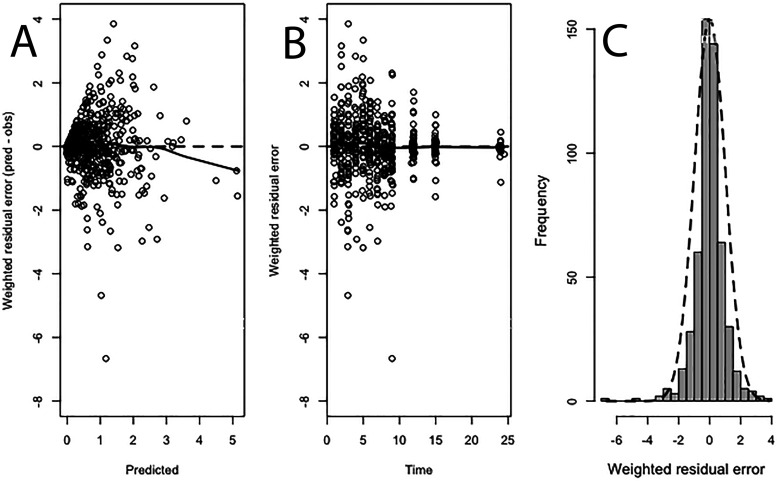
Residual plots for plasma concentrations. The average residuals did not vary from zero; *P* = 0.88 for weighted residual error versus predicted concentrations (A) and for weighted residual error versus time (B). The solid line in panels A and B is the loess regression. The residuals were normally distributed as assessed using D’Agostino, Shapiro-Wilk, and Kolmogorov-Smirnof tests (C). *P* > 0.05.

The PK of PZQ in plasma and breast milk was comodeled using a population methodology with the program Pmetrics (15). A standard 3-compartment PK model consisting of an absorptive compartment (i.e., gut), central compartment (i.e., bloodstream) and peripheral compartment (i.e., rest of the body) was initially fitted to the data before the potential impact of covariates on the PK was assessed. The mean, median, and dispersions for the population PK parameters from the base model are summarized in Table 2. The fit of the model to the data was acceptable. There was an acceptable degree of bias and imprecision as determined by a normalized prediction distribution error (NPDE) analysis for both plasma and breast milk concentrations (data not shown). The observed-predicted values are shown in Fig. 3, and the individual plots are shown in Fig. S1 in the supplemental material. The residuals are shown in Fig. 4. The mean of the weighted residuals was not statistically different from zero, and the weighted residuals were normally distributed. The Bayesian posterior estimates for each patient were calculated, and these were used to assess the impact of covariates on the PK as well as to estimate drug exposure of PZQ in each patient.

**TABLE 2:** Parameter values from the population PK model

Parameter (units)^*a*^	Mean	Median	SD	CV (%)^*b*^
Ka (h^–1^)	2.012	0.395	4.301	213.750
SCL/F (liter/h)	324.075	277.447	175.373	54.115
Vc/F (liter)	183.006	142.618	93.211	50.933
Kcp (h^–1^)	19.313	18.941	10.167	52.644
Kpc (h^–1^)	15.816	13.996	9.447	59.733
Kcb (h^–1^)	18.750	19.301	9.387	50.067
Kbc (h^–1^)	17.816	17.077	7.845	44.031
Vb/F (liter)	612.130	563.802	395.661	64.637
Lag (h)	0.772	0.868	0.233	30.202

a Parameters are as follows: Ka is the first-order absorption constant; SCL/F is the apparent clearance; Vc/F and Vb/F are the apparent volumes of the central and breast compartments, respectively; Kcp, Kpc, Kbc, and Kcb are the first-order intercompartmental rate constants; lag is the delay between drug administration and the appearance of drug in the central compartment.

b CV, coefficient of variation.

**FIG 5:**
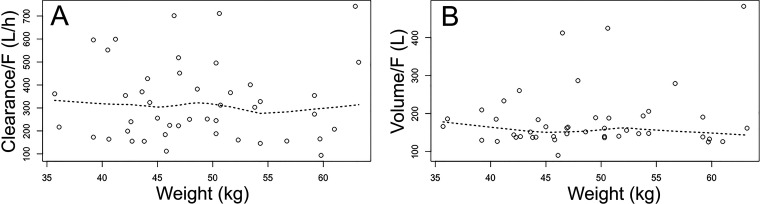
(A and B) The relationship between weight and clearance/F (A) and weight and volume/F (B). The volume is the volume of the central compartment. Neither relationship is statistically significant, with *r* = 0.03 (*P* = 0.84) and 0.16 (*P* = 0.29) for clearance/F and volume/F, respectively. The broken line is the loess line.

There was no relationship between weight and Bayesian estimates for the apparent clearance (i.e., clearance/F) or between weight and the apparent volume of the central compartment (i.e., V/F) (Fig. 4). The correlation coefficient for these relationships was *r* = 0.0303 (95% confidence interval [CI], –0.2656 and 0.3210; *P* = 0.8435) and *r* = 0.1617 (95% CI –0.1384 and 0.4346; *P* = 0.2885), respectively. Hence, covariates were not incorporated into the structural model (Fig. 5).

**FIG 6:**
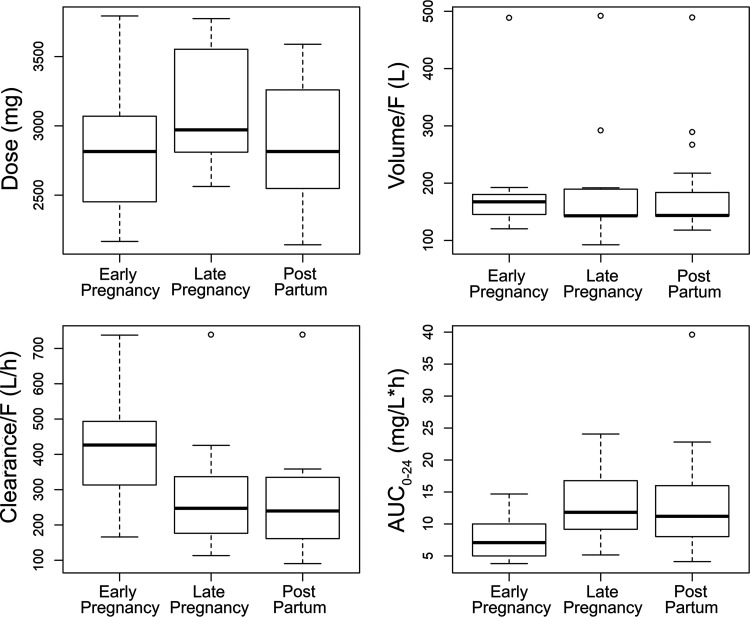
(A to D) Box plots showing the relationship between various stages of pregnancy and dose (A), volume of the central compartment/F (B), clearance/F (C) and the area under the concentration-time curve (AUC_0–24_) in panel D. There was no relationship between the stage and pregnancy and the absolute dose (mg) and volume/F (*P* = 0.2072 and 0.626, respectively). Women in the early pregnancy group have a higher clearance/F than other women (*P* = 0.016 for all groups) and a lower AUC_0–24_ (*P* = 0.01 for all groups).

There were no differences in the absolute dosage received by women within the three study groups (*P* = 0.21, Kruskal Wallis test; Fig. 6A). Furthermore, there was no relationship between the Bayesian estimates for the apparent volume of the central compartment and the study groups (Fig. 6B). However, there was a significant relationship between the apparent clearance and the stage of pregnancy. Women in the early-pregnancy group had higher apparent clearances than those in the other groups (Fig. 6C). Women in the early-pregnancy group had faster apparent clearance of PZQ than the postpartum women (*P* = 0.02). There were also differences between early- and late-stage pregnancy that approached, but did not achieve, statistical significance (*P* = 0.056).

Women in the early-pregnancy group had significantly lower AUC_0–24_ values than those in the late-pregnancy group (*P* = 0.0144) and the postpartum group (*P* = 0.0378). Since there were no differences in absolute dosage received by women in these groups, the lower AUC_0–24_ in early pregnancy can only be explained by the faster clearance that was observed in this group or by lower oral bioavailability. There were significant differences in the observed maximum concentration of drug in serum (*C*_max_) values between the groups. The overall differences were statistically significant using analysis or variance (ANOVA) (*P* = 0.019). There was a difference between the early- and late-pregnancy groups (*P* = 0.017 after Bonferroni correction) but not between early-pregnancy and postpartum women (*P* = 0.236) or late-pregnancy and postpartum women (*P* = 0.814). Further evidence of the potential importance of oral bioavailability affecting drug exposure (i.e., AUC_0–24_) was obtained from the relationship between apparent clearance (SCL)/F and V/F. Both were highly correlated (*r *= 0.636; 45 observations; *P* < 0.001), suggesting that F may have an impact on both parameters. Given the uncertainty regarding the impact of altered oxidative metabolism versus oral bioavailability as an explanation for the lower AUC_0–24_, we did not further complicate the structural model that was fitted to the data.

### Population PK of PZQ in breast milk.

The concentration-time course of PZQ in breast milk was variable (Fig. 2). The elimination of drug in breast milk was similar to that of plasma. The AUC_plasma_:AUC_breast milk_ mean ± standard deviation (SD) calculated from the Bayesian posterior estimates was 0.36. ± 0.13 with a range in the 15 lactating women of 0.19 to 0.55. The average concentration in breast milk was 0.185 mg/liter (i.e., AUC_0–24_/24). Therefore, the estimated average ingestion of PZQ by a newborn infant that consumes 150 ml/kg of breast milk per day was approximately 0.028 mg/kg per day (i.e., 0.185 mg/liter · 0.15 liter/kg).

The elimination half-life of PZQ from breast milk was 1.90 h. For a lactating woman of average weight observed in this study receiving 60 mg/kg in two divided dosages of 30 mg/kg, the estimated PZQ concentration in breast milk 24 and 48 h postdose was 0.0004 mg/liter and 3 × 10^−7^ mg/liter, respectively. Hence, 24 h postdose, there is only 0.01% of the maximal concentration of PZQ in breast milk, and at 48 h, the concentrations of drug were negligible.

### Monte Carlo simulations.

**FIG 7:**
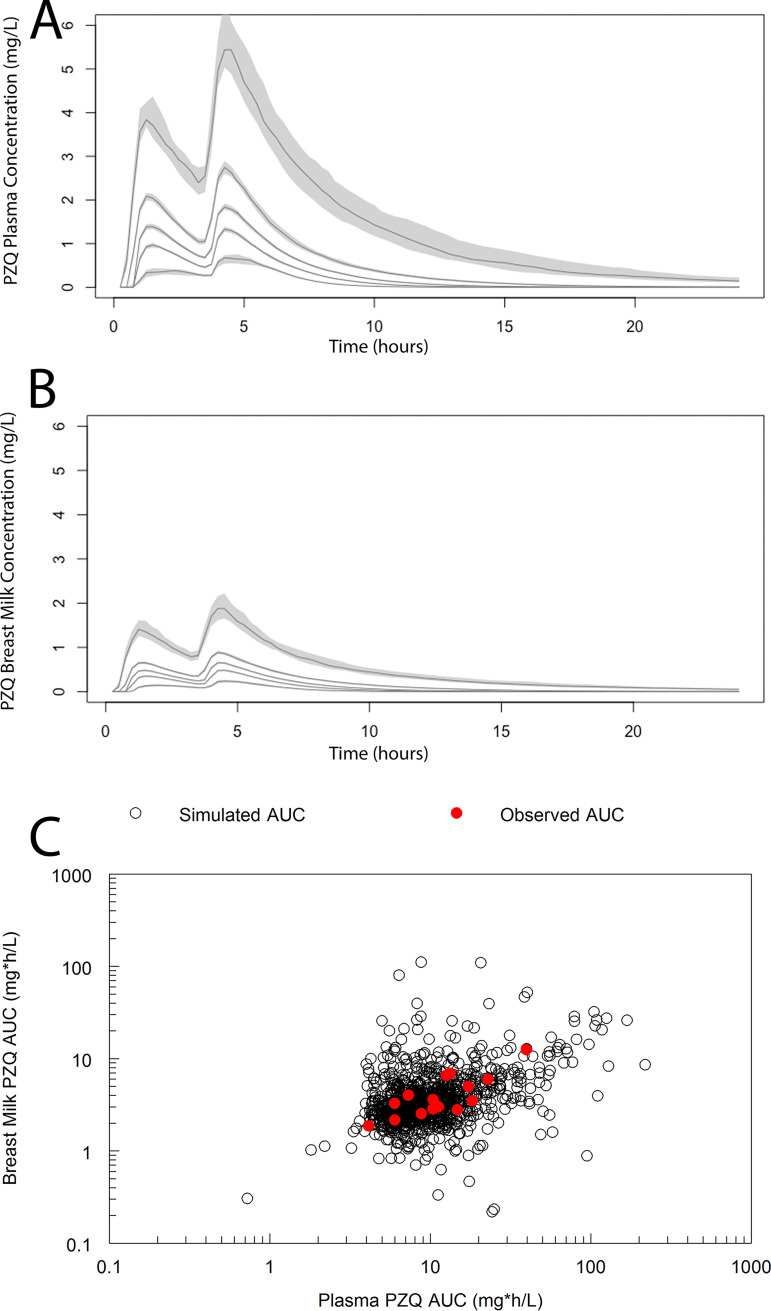
(A and B) Monte Carlo simulations showing the drug exposure in plasma (A) and breast milk (B) from 1,000 lactating women. The lines represent the 5th, 25th, 50th, 75th, and 95th centiles, and the gray shading represents the confidence interval around each centile. In panel C, the AUC_0–24_ in plasma versus breast milk in each simulated woman is shown with black open circles. The AUC_0–24_ from each of the 15 patients in the study are shown with solid red circles.

Monte Carlo simulations were performed using the median weight of study participants (47.9 kg). Pmetrics was used to generate a total of 1,000 lactating women. The concentration-time profile for each patient was determined. The 5th, 25th, 50th, 75th, and 95th centiles and their 95% confidence intervals bound in plasma and breast milk are shown in Fig. 7. The AUC_0–24_ in plasma and breast milk was calculated from the median Bayesian posterior estimates using the trapezoidal rule in the first 24 h following the initiation of therapy. A plot of the simulated AUCplasma versus AUCbreast milk is shown in Fig. 7 overlaid with the observed AUCs from the 15 lactating women in the study. The partitioning of PZQ into breast milk was comparable between the observed data and the simulations and was approximately 30%.

### Adverse events.

There were no severe adverse events documented in any of the women. Only two women had mild side effects with vomiting documented within 2 h of PZQ dosing and were excluded from PK analysis. The adverse events are summarized in Table 3.

**TABLE 3:** Numbers of adverse events by severity and cohort^*a*^

Reactogenicity	Cohort 1 (N = 17)				Cohort 2 (N = 15)				Cohort 3 (N = 15)			
	Severity				Severity				Severity			
	None (*n* [%])	Mild (*n* [%])	Moderate (*n* [%])	Severe (*n* [%])	None (*n* [%])	Mild (*n* [%])	Moderate (*n* [%])	Severe (*n* [%])	None (*n* [%])	Mild (*n* [%])	Moderate (*n* [%])	Severe (*n* [%])
Fever	16 (94.1)	0 (0)	1 (5.9)	0 (0)	13 (86.7)	2 (13.3)	0 (0)	0 (0)	13 (86.7)	1 (6.7)	1 (6.7)	0 (0)
Headache	9 (52.9)	6 (35.3)	2 (11.8)	0 (0)	9 (60.0)	5 (33.3)	1 (6.7)	0 (0)	6 (40.0)	8 (53.3)	0 (0)	1 (6.7)
Malaise	11 (64.7)	5 (29.4)	1 (5.9)	0 (0)	13 (86.7)	2 (13.3)	0 (0)	0 (0)	9 (60.0)	5 (33.3)	0 (0)	1 (6.7)
Abdominal pain	13 (76.5)	2 (11.8)	1 (5.9)	1 (5.9)	11 (73.3)	4 (26.7)	0 (0)	0 (0)	10 (66.7)	4 (26.7)	1 (6.7)	0 (0)
Nausea	5 (29.4)	11 (64.7)	1 (5.9)	0 (0)	9 (60.0)	6 (40.0)	0 (0)	0 (0)	8 (53.3)	6 (40.0)	1 (6.7)	0 (0)
Vomiting	11 (64.7)	5 (29.4)	1 (5.9)	0 (0)	12 (80.0)	3 (20.0)	0 (0)	0 (0)	14 (93.3)	1 (6.7)	0 (0)	0 (0)
Shortness of breath	16 (94.1)	1 (5.9)	0 (0)	0 (0)	14 (93.3)	1 (6.7)	0 (0)	0 (0)	15 (100.0)	0 (0)	0 (0)	0 (0)
Dizziness	10 (58.8)	7 (41.2)	0 (0)	0 (0)	13 (86.7)	2 (13.3)	0 (0)	0 (0)	12 (80.0)	3 (20.0)	0 (0)	0 (0)
Rash	15 (88.2)	2 (11.8)	0 (0)	0 (0)	13 (86.7)	2 (13.3)	0 (0)	0 (0)	13 (86.7)	1 (6.7)	1 (6.7)	0 (0)
Urticaria	16 (94.1)	1 (5.9)	0 (0)	0 (0)	15 (100.0)	0 (0)	0 (0)	0 (0)	14 (93.3)	1 (6.7)	0 (0)	0 (0)
Bloody stools	17 (100.0)	0 (0)	0 (0)	0 (0)	14 (93.3)	1 (6.7)	0 (0)	0 (0)	14 (93.3)	1 (6.7)	0 (0)	0 (0)
Any of the above symptoms	4 (23.5)	9 (52.9)	3 (17.6)	1 (5.9)	5 (33.3)	9 (60.0)	1 (6.7)	0 (0)	3 (20.0)	8 (53.3)	3 (20.0)	1 (6.7)

a *N*, number of subjects in population; *n*, number of subjects with at least one occurrence of an adverse event in the specified category.

## DISCUSSION

This is the first study to describe the PK of PZQ in pregnant and lactating women infected with *Schistosoma japonicum*. Women in early pregnancy had significantly lower AUC_0–24_ than women in late pregnancy and lactating postpartum women. The most likely explanation for the differences in clearance relate to pregnancy-induced increases in hepatic enzyme activity related to hormonal changes associated with pregnancy (9, 16). The absorption of PZQ is limited by grapefruit juice, suggesting the importance of oxidative mechanisms in the gut wall. PZQ is known to undergo high first-pass metabolism (6, 17). Estradiol and progesterone are both known to induce CYP3A4 in pregnancy (18) and are responsible for increased clearance of drugs such as midazolam (19, 20). However, these changes are typically more pronounced later in pregnancy, which is not consistent with the raw data or the estimates of clearance in this study. This observation raises that possibility that some of the changes may be related to differences in oral bioavailability in the study groups. There were differences in *C*_max_ between the groups (significantly lower in early pregnancy) and a high degree of correlation between SCL/F and V/F. It is possible that another pregnancy-related hormone or transporter expressed in early pregnancy has an impact on clearance and drug exposure.

We did not investigate the potential impact of hepatic metabolism on the PK variability of PZQ. Liver function may be potentially altered from schistosomiasis due to S. japonicum (21). The clearance of PZQ may also be affected by pharmacogenetic polymorphisms in CYP enzymes (e.g., CYP1A2, CYP3A4, CYP2B1, CYP3A5, and CYP2C19) and/or interactions with drugs or substances taken concomitantly that induce or inhibit specific isoenzymes of the CYP system (e.g., rifampin [22]). Several studies have reported a decrease in CYP1A2 (23) and estrogen inhibition of CYP2C19 (24) during pregnancy, requiring a dose adjustment of certain drugs (16).

The AUC_0–24_ is a measure of drug exposure (10) that has been used to link dosage with clinical outcomes in a recent PK/pharmacodynamic (PD) model in children with schistosomiasis in Uganda (25). In a recent study (24), the mean PZQ AUC_0–24_ values ranged from 8.2 to 14.6 mg · h/liter. These values are higher than the PZQ AUC_0–24_ mean estimated from 60 Ugandan children 3 to 9 years of age with intestinal schistosomiasis (2.71 mg · h/liter) (25). The relevance of this observation depends on whether the pharmacodynamics of PZQ against schistosomiasis in children and pregnant women are comparable. While women in early pregnancy have lower AUC_0–24_ than women in late pregnancy or postpartum, these values are significantly higher than those for children receiving a comparable dose for whom the efficacy of PZQ has been established. Hence, in principle, there does not appear to be any requirement to adjust the dosage according to the stage of pregnancy. However, further studies are required to document the clinical response in pregnant women with schistosomiasis.

There are limited studies on the partitioning of drugs into breast milk (26–29). A single previous study has examined PZQ concentrations in the breast milk of healthy lactating women (30). Our study provides further insights into the pharmacokinetics of PZQ in lactating women and the potential implications for mass drug administration programs. First, the amount of drug an infant ingests depends on the concentration of drug in breast milk. This changes rapidly over the initial 24 h postdose. The amount of drug that is ingested by an infant depends on the time of feeding relative to the administration of PZQ as well as the volume of milk that is consumed. Using estimates for an average concentration and volume of milk, the weight-based intake of 0.037 mg/kg is significantly less than that required for therapeutic efficacy (circa 40 to 60 mg/kg). Second, there is relatively little variability in the AUC in breast milk. We observed approximately a 2-fold variation in the 15 lactating women in this study, and the Monte Carlo simulations suggest that up to 10-fold variability may be expected if a larger number of women had been studied. Hence, the small amount from ingestion of breast milk is unlikely to be clinically relevant. The benefits of treating lactating women to prevent them from further developing schistosomiasis-related morbidity would seem to outweigh any potential risks. The PK data do not support the manufacturer’s suggestion to delay breastfeeding 72 h after taking PZQ (31).

Women have been systematically excluded from both studies and MDA efforts (14). We contend that pregnant and lactating women should not be excluded from any treatment efforts because of the demonstrated safety and efficacy of PZQ during gestation (12, 13). This study further demonstrates that there are unlikely to be clinically relevant pharmacokinetic differences in pregnant and lactating women. Untreated schistosomiasis may lead to more severe disease and chronic disability. For example, female genital schistosomiasis may lead to infertility and disruption of a healthy reproductive life (32). Women with intestinal schistosomiasis may have worsening anemia and liver fibrosis. Early treatment with PZQ is known to mitigate these late complications of schistosomiasis (33). A concern about the theoretical risks related to PZQ has led to pregnant women being excluded from mass drug administration programs (5); however, recent trials in pregnant women and this pharmacokinetic study suggest that the withholding of PZQ during pregnancy and lactation is not justified. (12, 13). Our PK results can help inform future drug efficacy studies in pregnant and lactating women with schistosomiasis.

## MATERIALS AND METHODS

### Study protocols and permissions.

The study was separately approved by the ethics review board of the Research Institute of Tropical Medicine in Manila, Philippines (number 2010-39), and the Institutional Review Boards from the Rhode Island Hospital in Providence, RI, USA (number 415810), Boston University Medical Center in Boston, MA, USA (number H30043), and the University of California at San Diego in San Diego, CA, USA (number 120559X). Informed consent was obtained from all study participants prior to enrollment.

### Study site and participants.

The study design is summarized in Fig. 1. Eligible patients that were living in villages in northeastern Leyte, Philippines, where S. japonicum is endemic, were identified and screened by local midwives. Patients with at least one positive stool sample for S. japonicum were then assessed by a study obstetrician at the Remedios Trinidad Romualdez (RTR) Hospital (Tacloban, Leyte, Philippines). The methodology for detection of parasites in stool is described elsewhere (12). Women were eligible if they met the following inclusion criteria: (i) infected with S. japonicum, (ii) aged 18 years or older, (iii) otherwise healthy as established by physician history, physical examination, and laboratory studies, (iv) had a normal obstetrical ultrasound, if pregnant, and (v) provided informed consent. Postpartum women were recruited from study villages; many had been considered for enrollment in the main RCT but were beyond the gestational age criteria. Eligibility criteria were the same as for pregnant women, with the exception of the criterion for pregnancy. Early pregnancy was defined as women in their 12th to 16th week of gestation, and late pregnancy as women in their 30th to 36th weeks of gestation.

### PZQ PK sampling.

Within 4 weeks of enrollment, patients received PZQ (Schering-Plough, Kenilworth, NJ, USA) in two dosages of 30 mg/kg administered approximately 3 h apart for a total dose of 60 mg/kg. Women were given local foods that consisted of a carbohydrate-rich snack prior to PZQ dosing, as this enhances absorption of the drug (7). After receiving PZQ, patients remained in the hospital for PK sampling and monitoring for adverse events. Patients were discharged approximately 24 h after the first dose. An indwelling venous catheter was placed to draw blood samples for assay for praziquantel concentrations, which were collected at the following times: for pregnant and postpartum women, prior to PZQ dosing and 1, 2, 3 (prior to administration of the 2nd dose of PZQ), 4, 5, 6, 7, 8, 9, 12, 15, and 24 h after the first dose of PZQ.

For lactating women, women hand-expressed breast milk, and samples were collected within 15 min of collection of the blood samples scheduled for 3, 6, 9, 12, 15, and 24 h after the first dose of PZQ. Blood samples for toxicity monitoring (complete blood count, blood urea nitrogen [BUN] and creatinine, liver function tests) were collected just before the first dose, 24 h after the dose, and at approximately 32 weeks gestation (early-gestation subjects only) or 10 to 14 days after the PZQ dose (late-gestation and lactating postpartum subjects). Newborns were monitored for clinical signs of toxicity until 28 days after delivery for early- and late-pregnancy subjects, with the final study visit at 28 days of life with the study pediatrician at RTR Hospital in Tacloban. Postpartum women were seen at RTR Hospital 2 weeks after administration of study drug.

Venous blood was drawn and samples were spun for 15 min at ∼5,000 × *g* and 20°C in an Eppendorf centrifuge. Plasma was removed and stored in two separate aliquots and at –80°C. Breast milk was stored at –80°C. Both plasma and breast milk samples were shipped on dry ice to the University of California at San Diego (UCSD) Pediatric Clinical Pharmacology Laboratory, where they were assayed for PZQ using high-performance liquid chromatography (HPLC)–electrospray mass spectrometry according to the methods of Bonato et al. (34). The lower limits of quantitation of the assay were 31.3 ng/ml for plasma and 4.3 ng/ml for breast milk.

### Quantification and resolution of PZQ and 4-OH PZQ in plasma and breast milk.

Praziquantel (PZQ) concentrations were quantified in plasma and breast milk with liquid chromatography mass spectrometry (LC/MS) using an Agilent liquid chromatograph/autosampler interfaced with a Sciex API 4000 mass spectrometer. Prior to analysis, proteins were removed from plasma/milk samples by precipitation with acetonitrile. Analytical-grade PZQ was obtained from Sigma-Aldrich. Separation of PZQ from other matrix constituents was obtained with an isocratic HPLC mobile phase consisting of 80% methanol and 20% formic acid (0.1%) in water, in conjunction with a 2.1 mm by 15 cm MacMod Ace-5 C18 reverse phase column. Mass transitions 313.2 → 203.1 served as quantification ions for PZQ detection, while mass transitions 313.2 → 174.1 served as qualification ion verification of PZQ. Quantitation was by means of external calibration using Analyst 1.6.1 software, with a qualification ion ratio threshold of ≤10% (deviation from expected). The dynamic range of the assay was 0.1 to 4,000 ng/ml and 2 to 2,200 ng/ml for plasma and breast milk, respectively. The precision of the assay was <11% and <15% at all calibration concentrations for plasma and breast milk, respectively. Assay accuracy was ≤±8% and ≤±13% for plasma and breast milk, respectively. Recovery from plasma was >91%, and recover was >87% for breast milk at all calibration concentrations.

### Population pharmacokinetics.

A population methodology was used to fit a structural model to the data. PZQ was allowed to redistribute back to the maternal plasma without terminal elimination via expression of breast milk. The model was structured in this way to avoid an unidentifiable solution, but also because the excretion of drug in breast milk was assumed to be minimal and the equilibrium was rapid. The structural model took the form

(1) XP(1)=Bolas−Ka×X(1)

(2) $$\mathrm{XP}(2)=\mathrm{Ka}\times X(1)-\frac{\left(\mathrm{SCL}\right)}{\mathrm{Vc}}\times X(2)+\mathrm{Kpc}\times X(3)+\mathrm{Kbc}*X(4)-\mathrm{Kcb}*X(2)$$

(3) XP(3)=Kcp×X(2)−Kcp×X(3)

(4) XP(4)=−Kbc*X(4)−Kcb*X(2)

where XP(1), XP(2), XP(3), and XP(4) are the rate of change of PZQ mass in the gut, central compartment, peripheral compartment, and breast milk, respectively. Similarly, X(1), X(2), X(3), and X(4) represent the mass (mg) of PZQ in the respective compartments. Bolus refers to the oral administration of PZQ; SCL is the first-order clearance of PZQ from the central compartment, Vc is the volume of the central compartment; Kpc, Kcp, Kcb and Kbc are the first-order intercompartmental rate constants. A lag function (not shown in the differential equations) was applied between the oral administration of PZQ and the appearance of drug in the central compartment.

The output equations were given by

Y(1)=X(1)/Vc (for the plasma concentrations)

Y(2)=X(4)/Vb (for the concentrations in breast milk)

where Vb is the volume of breast milk compartment.

The fit of the model to the data was informed by a linear regression of observed-predicted values before and after the Bayesian step, the log likelihood ratio, and a normalized prediction distribution error. The latter was used in place of a more traditional visual predictive plot because women received different dosages of PZQ. Both the mean and median parameter values were interrogated to see which measure of central tendency better described the data. Weighted residuals were calculated and plotted against predicted concentrations and time and assessed for normality using D’Agostino, Shapiro-Wilk, and Kolmogorov-Smirnof tests. Drug exposure was quantified in terms of the AUC_0–24_ as previously described by us (25). This was estimated using the trapezoidal rule using Pmetrics and estimated from the Bayesian posterior estimates from each study patient or from simulated patients.

### Statistical modeling.

The Bayesian estimates for clearance and AUC_0–24_ were modeled for study groups using univariate analysis of variance (ANOVA). Since both Bayesian estimates for clearance and AUC_0–24_ were not distributed normally, they were fitted on a natural log scale. The estimated means of clearance and AUC_0–24_ between individual study groups were compared in a *post hoc* analysis using Tukey’s test, and the reported *P* values were corrected for multiple comparisons.

## Supplementary Material

Supplemental file 1
