# Supplementary material for: Population Pharmacokinetics of Praziquantel in Pregnant and Lactating Filipino Women Infected with Schistosoma japonicum
Source: Antimicrob Agents Chemother. 2020 Aug 20;64(9):e00566-20. doi: 10.1128/AAC.00566-20 (PMC7449211; doi:10.1128/AAC.00566-20)
Supplement: Supplemental file 1 [file AAC.00566-20-s0001.pdf]

Supplementary figure 1: Observed/Predicted individual plots. Black line represents plasma levels of praziquantel. Red line represents breast milk levels and red crosses are the observed time points for the subset of women with breast milk data.

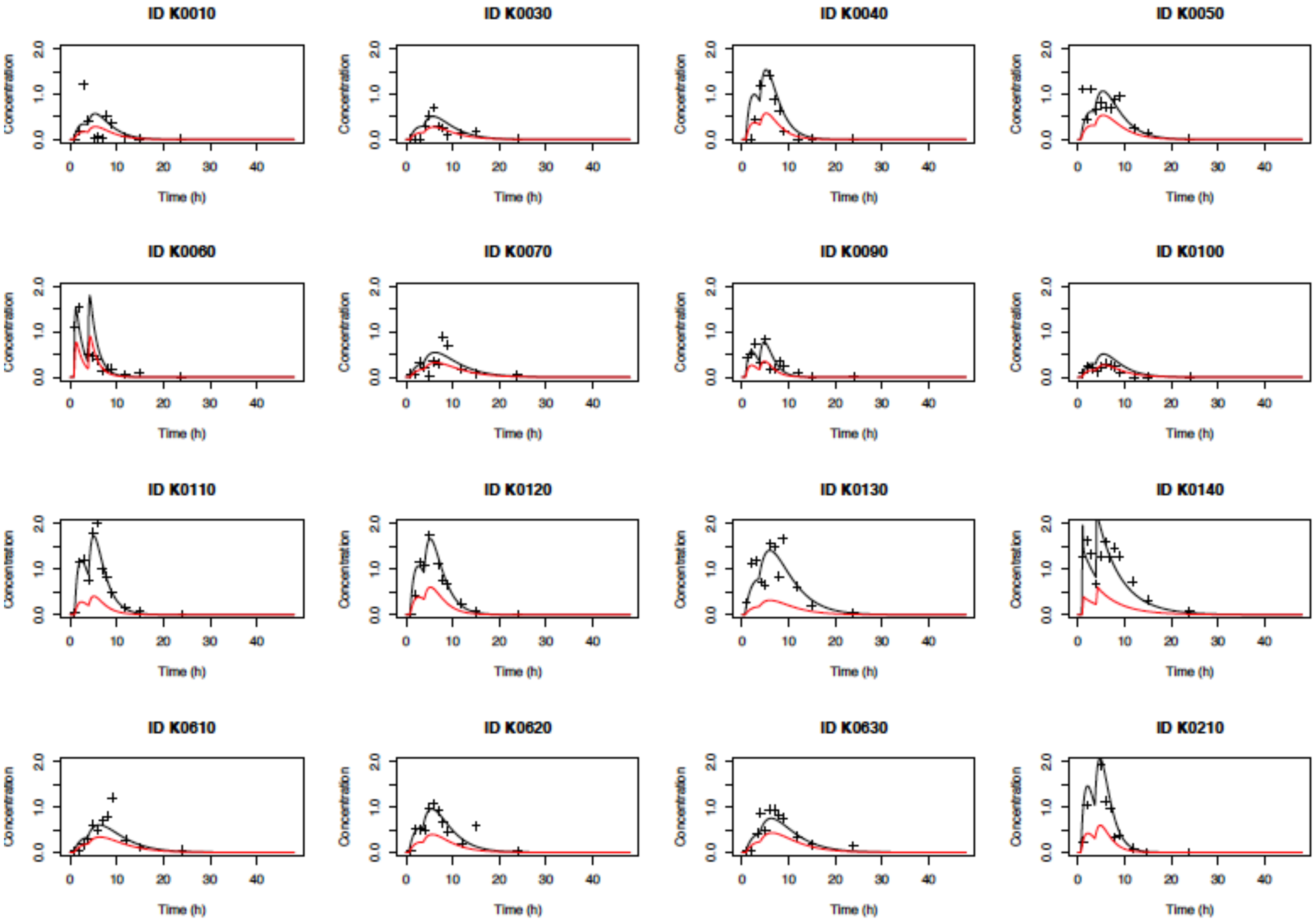

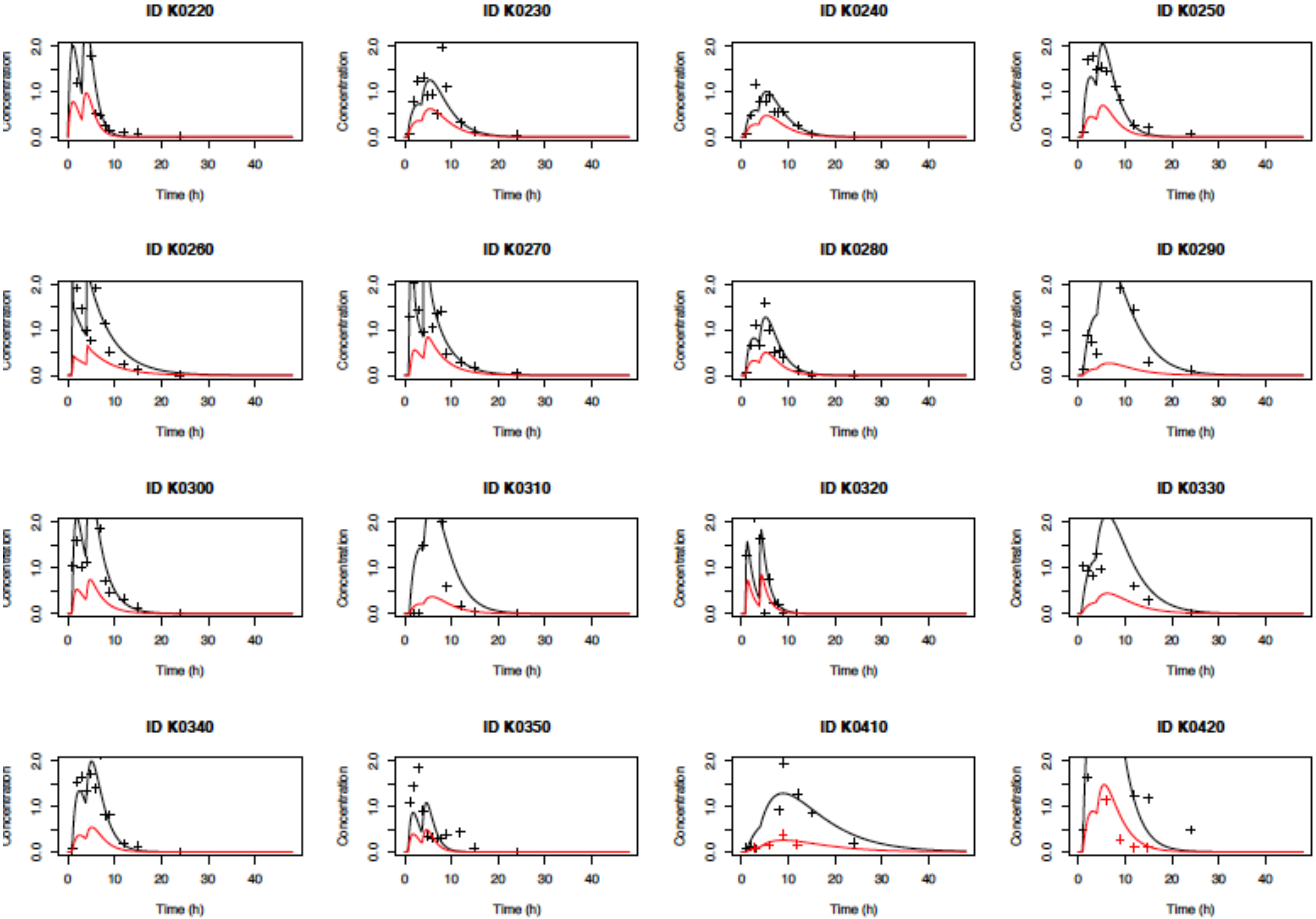
ID K0220
ID K0230
ID K0240
ID K0250
ID K0260
ID K0270
ID K0280
ID K0290
ID K0300
ID K0310
ID K0320
ID K0330
ID K0340
ID K0350
ID K0410
ID K0420
Concentration
0.0
1.0
2.0
Time (h)
0
10
20
30
40

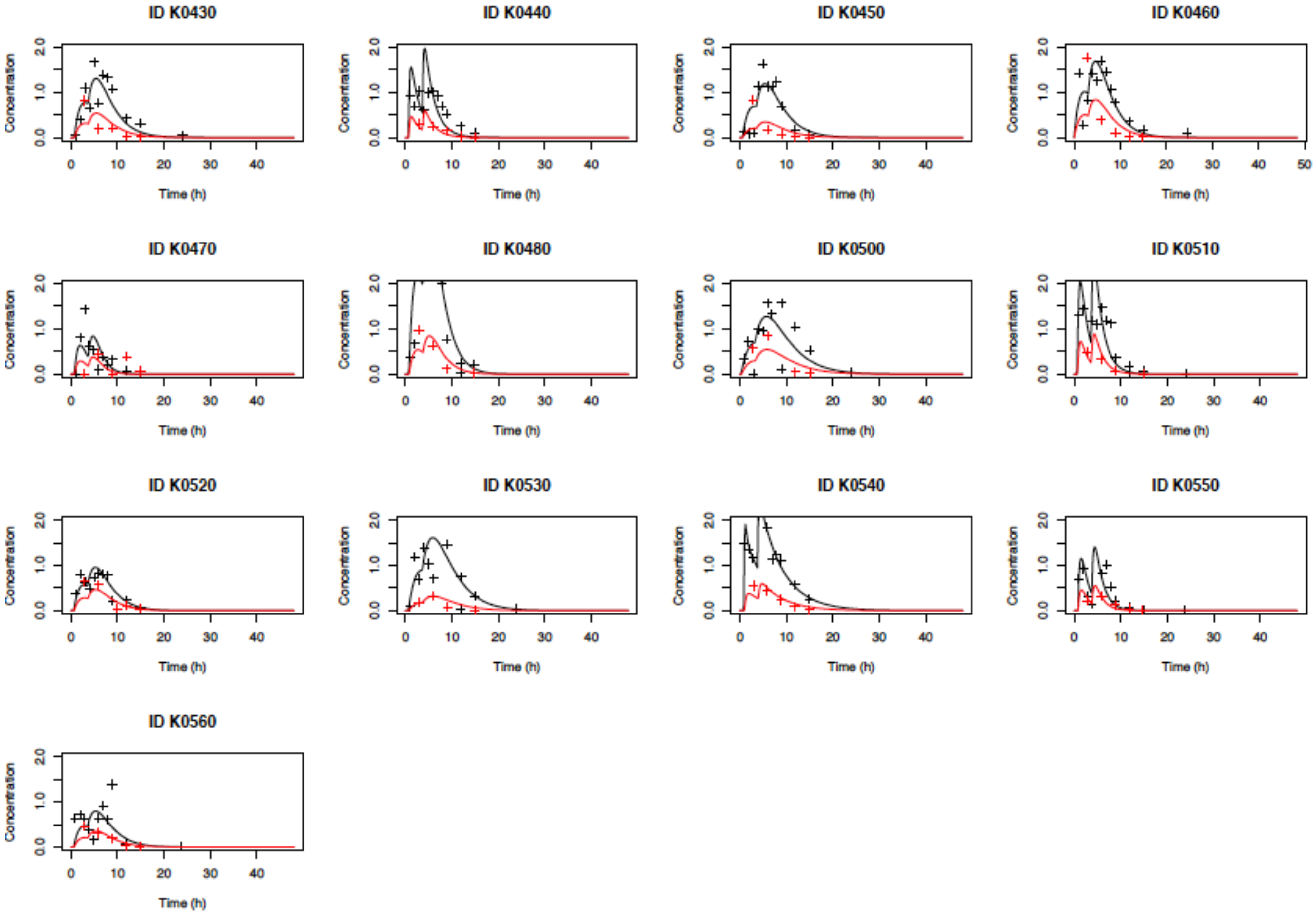
ID K0430
ID K0440
ID K0450
ID K0460
ID K0470
ID K0480
ID K0500
ID K0510
ID K0520
ID K0530
ID K0540
ID K0550
ID K0560
Concentration
Time (h)
